# Blinatumomab may induce graft versus host leukemia in patients with pre‐B ALL relapsing after hematopoietic stem cell transplant

**DOI:** 10.1002/ccr3.604

**Published:** 2016-06-24

**Authors:** Muhammad Waqas Khan, Zartash Gul

**Affiliations:** ^1^ Department of Internal medicineDivision of Hematology/Bone Marrow TransplantationUniversity of KentuckyLexingtonKentucky

**Keywords:** BiTe antibody in ALL, blinatumomab in pre‐B ALL, post transplant ALL management, refractory ALL treatment

## Abstract

Blinatumomab, a bispecific T‐cell engager monoclonal antibody used to manage Philadelphia chromosome‐negative relapsed or refractory B‐cell precursor acute lymphoblastic leukemia (ALL) can be used to treat patients by inducing graft versus leukemia reaction post allogeneic hematopoietic stem cell transplantation, a feature which it was post allogeneic bone marrow transplantation, a feature which this drug was not aimed to do.

## Case

A 61‐year‐old female was diagnosed with Pre‐B ALL (MLL positive, mixed lineage leukemia) CNS negative, relapsed after HyperCVAD and was refractory to Asparaginase, Vincristine, Dexamethasone (Capizzi regimen) followed by Cytoxan/Etoposide (Cytoxan dose 50 mg/kg intravenous on days 3–5; Etoposide (VP16) dose 600 mg/m^2^ intravenous every 8 h, with four doses started on day 2 of treatment). She achieved remission with 9 *μ*g dose Blinatumomab; a grade 3 neurological toxicity is usually seen with 28 *μ*g doses (Table 1). She was subsequently transplanted in molecular remission from a matched sibling donor using Busulfan (AUC 4800) and Fludarabine (30 mg/m^2^ on days 1–5). She received Tacrolimus, Methotrexate, and Rituximab for GVHD prophylaxis (graft versus host disease). On the 100th evaluation day, she relapsed with a loss of donor chimerism to 43% without evidence of GVHD.

**Table 1:** Grading of chemotherapy‐induced peripheral neuropathy

Scale	Grade 0	Grade 1	Grade 2	Grade 3	Grade 4
ECOG 11	None	Mild paresthesisas Decreased deep tendon reflex	Severe paresthesias Absent deep tendon reflex	Disabling sensory loss Severe peripheral neuropathic pain Bladder dysfunction	Respiratory dysfunction secondary to weakness Paralysis
WHO 12	None	Paresthesias Decreased tendon reflexes	Severe paresthesias Mild weakness	Intolerable paresthesias Marked motor loss	Paralysis

**Figure 1:**
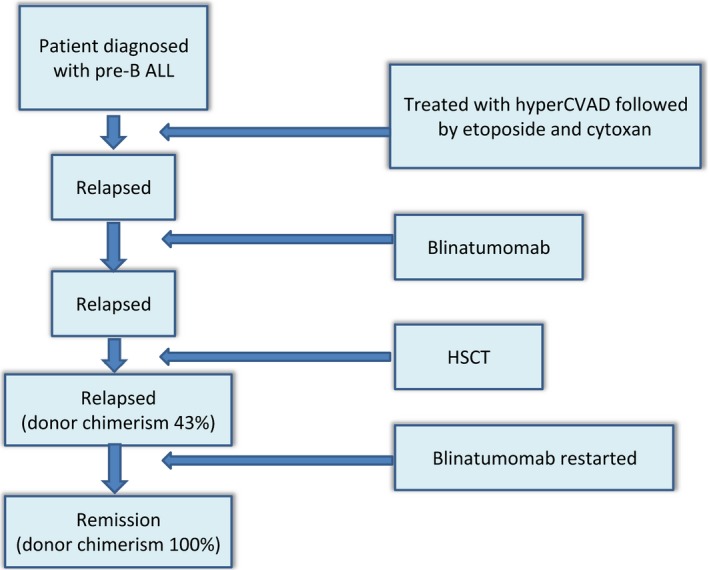
Timeline indicating sequence of events.

Blinatumomab was restarted at lower dose of 9 *μ*g and molecular remission was achieved. It was held after two cycles because she developed nausea, diarrhea, and elevated liver enzymes (ALT‐820U/L; ALP‐243U/L). It was noted that she had a 100% donor chimerism and the biomarkers for GVHD had increased especially REG3 alpha (Regenerating islet‐derived protein 3 alpha‐a gene encoding pancreatic secretory protein that is involved in cellular differentiation and proliferation) that increased to 217 ng/mL. She was started on prednisone at 1 mg/Kg (25 mg daily) which resulted in resolution of her symptoms and decrease in levels of REG 3 alpha (88 ng/mL [Normal <74 ng/mL]). She gained weight and her liver enzymes reduced to near normal (ALT 67U/L). Prednisone was tapered to 10 mg PO daily. She is currently day 240 post‐transplant and is in remission with a 100% donor chimerism (Fig. 1).

## Discussion

Adult acute lymphoblastic leukemia remains a challenging disease to treat with precursor‐B ALL comprising nearly 80% of cases 1.

This aggressive lymphoid malignancy comprises of the replacement of the cells present in the bone marrow compartment with blasts cells. Although ALL may have several phenotypic presentations, precursor B‐cell (pre‐B) ALL is the most common phenotype present 1. Multidrug chemotherapy regimens followed by a consolidation phase with high‐dose chemotherapy is the initial stage of treatment during the management of this disease. A second intensive regimen is often administered, which is generally followed by a few years of low‐dose maintenance chemotherapy in those not proceeding to allogeneic hematopoietic stem cell transplant (HSCT).

The CD19 antigen is expressed in almost all precursor‐B ALL patients, hence representing an interesting target for therapeutic research. Blinatumomab, a bispecific T‐cell‐engaging (BiTE) monoclonal antibody engages polyclonal T cells to CD19‐expressing B cells by binding to both CD3 and CD19. It brings them in close quarters to the malignant B cells, potentiating T‐cell‐induced cytotoxic activity 2, 3. BiTE antibodies are genetically constructed single chain antibodies that use a recombinant linked nonimmunogenic five‐amino acid chain that combines two variable regions of a normal antibody with different specificities (scFvs [single‐chain variable fragment] on CD19 and CD3 on T cells) 3. This connector allows a high degree of flexibility in rotation that will be needed for binding each of the CD3 and CD19 epitopes on cell membranes. The polyclonal T‐cell population creates an antitumor response 3. BiTE antibodies direct a T‐cell cytotoxic response by not targeting the major histocompatibility complexes which are often downregulated on tumor cells, regardless of their tumor immune escape mechanisms.

Blinatumomab was initially evaluated in B‐cell non‐Hodgkin's lymphoma (NHL) and in B‐cell ALL 4, 5. Cytokine‐release syndrome (CRS), a known adverse event with blinatumomab therapy is usually characterized by fevers, chills, and hypotension that may or may not be associated dyspnea in severe cases. This syndrome is due to the rapid malignant cell destruction by T lymphocytes during the initial infusion. Fever may be seen in up to 70% of the patients treated 4, 5. Pretreatment with steroids decreases the severity of this syndrome. Central nervous system events (CNS‐seizures and encephalopathy) have also been reported in almost 20% of patients, though all CNS events were reversible upon withholding the drug 5. Hypogammaglobulinemia, leukopenia with neutropenia, and B‐cell lymphocytopenia may frequently be encountered in patients treated with blinatumomab 6.

In a study by Topp et al. 7 in 2012, 36 patients with relapsed or refractory B‐cell ALL in a phase II trial were evaluated. Three groups were evaluated in an intrapatient dose escalation manner with an optimal determined dosage of 5 *μ*g/m^2^/24 h in week 1, and 15 *μ*g/m^2^/24 h on subsequent weeks and cycles. CNS events and CRS were common at higher doses during initial treatment due to the high tumor burden 7. Hematologic complete remission (CR) was achieved in 72% (26 patients). CR was more common in patients with first relapse (95% CR rate; 20 out of 21 patients) than those treated after subsequent relapse (40% CR rate; six out of 15 patients). Allogeneic hematopoietic stem cell transplant (HSCT) was performed in 13 of the 26 responders with a median follow‐up 10.7 months 7. Overall, the median survival was 9.8 months, with a 14.1‐month median survival in responders. In comparison with the median survival of 24 weeks in relapsed ALL with chemotherapy, blinatumomab demonstrated superior efficacy 8.

We hypothesize that suppression of CD 19‐positive B cells may induce suppression of B regulatory cells which in turn may lead to decrease T regulatory cells, thus leading to graft versus leukemia (GVL) effect, manifesting as GVHD 9, 10. We suggest that blinatumomab might play a role in maintenance therapy after allogeneic HSCT and warrants prospective assessment.

## Conclusion

In summary, we would like to report a case of pre‐B ALL with aggressive course refractory to multiple lines of therapy including hematopoietic stem cell transplant that responded to blinatumumab, resulting in complete donor chimerism and induction of GVHD that responded to steroids and remains quiescent with steroid taper. From our literature search, this is the first relapsed patient reported after allogeneic HSCT that attained full donor chimerism, induction of GVL as manifested by mild GVHD, and complete remission with blinatumomab which shows potential of this strategy as maintenance therapy after allogeneic HSCT in this patient population.

## Conflict of Interest

None declared.
